# Metabolomics Reveals Effect of Zishen Jiangtang Pill, a Chinese Herbal Product on High-Fat Diet-Induced Type 2 Diabetes Mellitus in Mice

**DOI:** 10.3389/fphar.2019.00256

**Published:** 2019-03-19

**Authors:** Jianping Chen, Lin Zheng, Zhaoliu Hu, Fochang Wang, Shiying Huang, Zhonggui Li, Ping Zheng, Shangbin Zhang, Tiegang Yi, Huilin Li

**Affiliations:** ^1^Shenzhen Key Laboratory of Hospital Chinese Medicine Preparation, Shenzhen Traditional Chinese Medicine Hospital, Shenzhen, China; ^2^The Fourth Clinical Medical College of Guangzhou University of Chinese Medicine, Shenzhen, China

**Keywords:** herbal medicine, type 2 diabetes, metabolomics, potential biomarkers, metabolic pathways

## Abstract

A Chinese herbal decoction, Zishen Jiangtang Pill (ZJP), has been clinically prescribed to diabetic patients to prevent excessive blood sugar levels for decades. However, the potential mechanisms of this action have not been well investigated. The purpose of this study was to explore the metabolic variations in response to ZJP treatment for an animal model of obese type 2 diabetes. An UHPLC-Orbitrap/MS-based metabolomics tool was conducted to reveal the potential mechanisms of ZJP on diabetic mice. The treatment with ZJP significantly restored the increased levels of insulin, glucose and total cholesterol in high-fat diet mice. A total of 26 potential biomarkers were found and identified in serum samples, amongst which 24 metabolites were robustly affected and driven back to the control-like levels after ZJP treatment. By analyzing the metabolic pathways, glutathione metabolism, steroid hormone biosynthesis and glycerophospholipid metabolism were suggested to be closely involved in diabetes disease. From the above outcomes, it can be concluded that ZJP exhibits a promising anti-diabetic activity, largely due to the regulation of phospholipid metabolism, including phosphatidylcholines, lysophosphatidylcholines, and phosphatidylinositol.

## Introduction

Type 2 diabetes mellitus (T2DM) is the most common form of diabetes mellitus and accounts for around 95% of all diabetes cases worldwide (Palanisamy et al., 2018). The number of people suffering from T2DM have rapidly boosted over recent decades, which has become an expanding global health problem (Einarson et al., 2018; Ma et al., 2018). A better understanding of the key factors contributing to T2DM is critical for determining future prevention and intervention programs. Insulin resistance and inadequate insulin secretion remain the core defects in T2DM (Lowe and Bain, 2013; DeFronzo et al., 2015). Many newer anti-diabetic drugs have been developed and introduced in to the market in recent years to treat diabetic patients with hyperglycemic conditions, yet many of these drugs have potential side effects (Palanisamy et al., 2018). Recently, T2DM patients also may choose alternative therapeutic options, such as traditional Chinese medicine (TCM). The multiple pathogenetic disorders relating to T2DM dictate that multiple anti-diabetic drugs, used in combination, will be required to control normoglycaemia. TCM usually involves in a combination of various herbs as a decoction to achieve the therapeutic activities and is becoming increasingly popular and recognized throughout the world (Wei and Zheng, 2008; Zhou et al., 2016). Hence, TCM may be a source of great interest in developing effective and safe agents to improve quality of life in diabetic patients.

Zishen Jiangtang Pill (ZJP), one of TCM products, consists of Astragali Radix, Rehmanniae Radix Praeparata, Epimedii Folium, Notoginseng Radix et Rhizoma and other herbs. ZJP has been clinically prescribed for T2DM patients with good effectiveness to maintain blood glucose level for decades, as it is considered to possess the efficacies of tonifying qi and yin, nourishing kidney and bones (Li et al., 2018). The previous pharmacological studies have showed that ZJP could decrease the blood glucose concentration in diabetic rats (Li et al., 2018). However, the underlying mechanism of ZJP on diabetes is less known.

Within the last decade, metabolomics seeks to develop the systematic analysis of all small molecule metabolites and has been successfully applied to decipher molecular mechanisms of TCM (Cao et al., 2015; Li et al., 2017; Wang et al., 2017). Metabolomics is a holistic profiling of small molecule metabolites offering a snapshot of physiological processes. The holistic view employed by metabolomics is similar to that of TCM, which allows us to deeply investigate TCM with complex conditions and multiple factors (Wang et al., 2015). The recent emerging development in mass spectrometry technologies provides an analytical platform in understanding the pathophysiology of diabetes and its complications (Sas et al., 2015).

Here, in our current study, we used metabolomics tools to investigate the role of ZJP in type 2 diabetic mice induced by high-fat diet and its potential mechanism. An UHPLC-Orbitrap/MS-based metabolomics approach was developed to identify the metabolic profiles of T2DM model and normal mice, and the potential biomarkers for T2DM were screened. Then the effects of ZJP on these biomarkers and its corresponding metabolism pathway were also analyzed.

## Materials and Methods

### Chemical and Reagent

Zishen Jiangtang Pill was provided by Pharmaceutical Department of Shenzhen Traditional Chinese Medicine Hospital (Lot no. 20170509). ZJP consists of night herbs and the mixed proportion of respective herb is illustrated in Table 1. All the botanical names can be checked and validated using http://mpns.kew.org/mpns-portal/?_ga=1.111763972.1427522246.145907734. Herbs used in this study were obtained from Shenzhen Huahui Pharmaceutical Co., Ltd. The plant materials were authenticated by Prof. Shangbin Zhang based on their morphological characteristics. The voucher specimens were deposited in the herbarium of Pharmaceutical Department, Shenzhen Traditional Chinese Medicine Hospital. Assurance of quality control for all the materials was validated according to the Chinese Pharmacopoeia (China Pharmacopoeia Committee, 2015). ZJP was prepared based on the manufacturing process of the local FDA in China. In brief, Astragali Radix and Notoginseng Radix et Rhizoma were prepared together as a superfine powder. The rest seven herbs of ZJP were weighed and extracted in boiling water twice for 1 h. After centrifugation, the supernatant was concentrated under reduced pressure to concentrated solution, and mixed with aforesaid powder to form into pills. The yield of the extract was ∼31.9%. HPLC-MS was conducted to confirm the quality of the ZJP, as indicated in Supplementary Figure 1. Deltamine was purchased from Shiguibao (Shiguibao, China). HPLC grade acetonitrile and formic acid were purchased from TEDIA (Fairfield, OH, United States). Deionized water was prepared using a MilliQ Ultrapure water system (Millipore, Merck, Germany). All other reagents used here were analytical grade.

**Table 1:** The composition and proportion of herb in ZJP.

Botanical name	Herbal name	Chinese name	Dosage
*Astragalus membranaceus* (Fisch.) Bge. var. *mongholicus* (Bge.) Hsiao	Astragali Radix	Huang-Qi	25 g
*Rehmannia glutinosa* Libosch.	Rehmanniae Radix Praeparata	Shu-Di-Huang	25 g
*Epimedium brevicornu* Maxim.	Epimedii Folium	Yin-Yang-Huo	25 g
*Panax notoginseng* (Burk.) F. H. Chen	Notoginseng Radix et Rhizoma	San-Qi	12 g
*Codonopsis pilosula* (Franch.) Nannf.	Codonopsis Radix	Dang-Shen	20 g
*Achyranthes bidentata* Bl.	Achyranthis Bidentatae Radix	Niu-Xi	20 g
*Schisandra chinensis* (Turcz.) Baill.	Schisandrae Chinensis Fructus	Wu-Wei-Zi	12 g
*Polygonatum kingianum* Coll. et Hemsl.	Polygonati Rhizoma	Huang-Jing	20 g
*Drynaria fortune* (Kunze) J. Sm.	Drynariae Rhizoma	Gu-Sui-Bu	20 g

### Induction of Experimental Diabetes Mellitus in Mice

Six-week-old male C57BL/6 mice were purchased from Nanjing University-Nanjing Biopharmaceutical Institution. Experimental animals were maintained in a specific pathogen-free (SPF) animal facility under a 12 h light-dark cycle, with food and water *ad libitum*. This study was carried out in accordance with the recommendations of National Institutes of Health Guideline for the care and use of laboratory animals. All animal procedures were conducted with protocol approval from the Ethics Committee of Shenzhen Traditional Chinese Medicine Hospital, Guangzhou University of Chinese Medicine (Shenzhen, China), and all efforts were made to minimize animal suffering.

Mice were provided either normal diet or high-fat diet for 12 weeks. The control group was fed with normal diet (Code LAD3001M, Nantong Trofee Feed Technology Co., Ltd.). The experimental diet was a modified model based on the Van Heek series 60% high-fat obesity model diet (Code TP23400, Nantong Trofee Feed Technology Co., Ltd.), and the feed was produced according to the AIN93 standard. After about 12 weeks of modeling, high-fat diet mice were randomly divided into 4 groups (*n* = 10 per group): Group 1: model group (high-fat diet only); Group 2: Deltamine group (168 mg/kg/d); Group 3: high dose of Zishen Jiangtang Pill (H-ZJP) group (1.51 g/kg/d); Group 4: low dose of Zishen Jiangtang Pill (L-ZJP) group (0.75 g/kg/d). The same volume of distilled water was given to control and model groups. After 8 weeks treatment, mice were sacrificed. Blood samples were collected from retro-orbital veins.

### Biochemical Analysis

After drug administration for 8 weeks, oral glucose tolerance test (OGTT) and insulin tolerance test (ITT) were measured (Chaput et al., 2007; Pacifici et al., 2014). Briefly, OGTT was performed in 12-h fasted mice by measuring tail blood glucose after oral glucose administration (2 g/kg) by gavage at 0, 30, 60, and 90 min. ITT was performed using intraperitoneal injection of 0.75 international units (I)/kg insulin in mice fasted for 4 h, and glucose levels were measured after 0, 15, 45 and 75 min. Blood samples were obtained from the tail vein using an automated OneTouch Glucometer (LifeScan, Milpitas, CA, United States). OGTT was measured 4 days before ITT. 2 to 3 days after ITT, the animals were sacrificed. Fasting blood glucose (FBG), fasting blood insulin (FINS), blood triglycerides concentration (TG) and blood total cholesterol concentration (TC) of each group were tested by glucose assay kit (Nanjing Jiancheng Bioengineering Institute, China), rat/mouse insulin ELISA (Merck, Germany), triglycerides assay kit (Nanjing Jiancheng Bioengineering Institute, China) and total cholesterol assay kit (Nanjing Jiancheng Bioengineering Institute, China). The insulin resistance index (HOMA-IR) and quantitative insulin sensitivity check index (QUICKI) were calculated. Calculation formula: HOMA-IR = (FBG, mmol/L) × (FINs, mmol/L)/22.5; QUICKI = 1 / [log (FINs μU/mL) + log (FBG mg/dL)].

### Immunohistochemistry Analysis

The islet tissues of mice were taken, fixed with 4% neutral paraformaldehyde, made into 4 μm paraffin sections after dehydration, embedding with wax, and slicing. The sections were incubated with specific primary antibodies directed against insulin (Cat No. ab8304, Abcam) and glucagon (Cat No. ab36598, Abcam), respectively. Subsequently, sections were incubated with HRP-conjugated secondary antibodies (Cat NO. KIT-9901, MXB Biotechnologies) and visualized with a fluorescence microscope (Olympus, Japan).

### Sample Preparation for LC-MS Analysis

Serum samples were thawed at room temperature, and 100 μl of which was added with 300 μl ice cold acetonitrile. The solution was thoroughly mixed by vortex for 30 s, kept at 4°C for 10 min, then centrifuged (12,000 rpm for 10 min at 4°C). The supernatant (250 μl) was transferred and added with 20 μl IS (2-Chloro-L-phenylalanine, 1 mg/ml), then evaporated to dryness under nitrogen gas at 37°C. The residue was re-dissolved with 100 μl 10% acetonitrile and centrifuged (13,000 rpm for 10 min at 4°C) after being vortexed for 60 s. The supernatant was transferred to auto-sampler vials for further UHPLC-Orbitrap/MS analysis.

### Method Validation

To guarantee the quality of the non-targeted bioanalytical data, quality control (QC) samples were used for method validation. Herein, 20 μl of serum samples from each group was pooled to obtain a QC sample, and QC samples were extracted using sample extraction method mentioned above. The QC specimens were analyzed every six samples throughout the whole analysis procedure. Repeatability and stability were performed by preparing and analyzing six QC samples.

Chromatographic separation was performed on a Dionex UltiMate 3000 UHPLC system (Thermo Scientific, San Jose, CA, United States). Waters Acquity BEH C18 column (2.1 × 100 mm, 1.7 μm, Waters, Milford, United States) was applied for all analyses at 30°C. The mobile phase was composed of 0.1% formic acid water (A) and acetonitrile (B) at a flow rate of 0.2 mL/min. The injection volume was set as 2 μl. For positive mode analysis, the gradient conditions were as follows: 0–4 min 10–13% B; 4–6 min 13–50% B; 6–10 min 50% B; 10–14 min 50–85% B; 14–18 min 85% B; 18–20 min 85–100%; 20–25 min 100%; re-equilibrate: 10 min. For negative mode analysis, the gradient conditions were as follows: 0–2 min 15% B; 2–4 min 15–55% B; 4–8 min 55% B; 8–14 min 55–70% B; 14–22 min 70–80% B; 22–24 min 80–100%; 24–28 min 100%; re-equilibrate: 10 min.

Mass spectrometry experiments were accomplished on an orbitrap mass spectrometer (LTQ ORBITRAP VELOS PRO, Thermo Fisher Scientific, San Jose, CA, United States) equipped with an electrospray ionization (ESI) source in both positive and negative modes. The scan range was from 100 to 1500. The MS parameters: electrospray ionization temperature (°C): 350 (ESI^+^)/350 (ESI^−^); Sheath Gas Flow Rate(arb): 35 (ESI^+^)/35 (ESI^−^); Aux Gas Flow Rate: (arb): 10 (ESI^+^)/10 (ESI^−^); Sweep Gas Flow Rate(arb): 0 (ESI^+^)/0 (ESI^−^); I Spray Voltage(kV): 3.2 (ESI^+^)/3.2 (ESI^−^); Capillary Temp(°C): 300 (ESI^+^)/300 (ESI^−^).

### Data Processing

Raw data files were pretreated, procedures included peak finding, alignment, filtering and normalization to total area, a three-dimensional data set consisted of sample information, peak intensities, peak retention time (RT) and the mass-to-charge ratio (m/z) was obtained. Retention time and the m/z data were used to be identifier for each ion. Moreover, peaks with missing value (ion intensity = 0) in more than 80% samples were removed in order to obtain consistent variables. Then the resultant data matrices were imported into the SIMCA14.1 (Umetrics, Umeå, Sweden) software for multivariate statistical analysis. The analysis methods containing principal components analysis (PCA), partial least-squares discriminant analysis (PLS-DA) and orthogonal partial least-squares discriminant analysis (OPLS-DA) were used for metabolite profile analysis (Chang et al., 2017).

### Biomarker Identification and Metabolic Pathway Analysis

Orthogonal partial least-squares discriminant analysis model was performed to visualize the metabolic difference between model group and control group. Those variables with VIP > 1 in the OPLS-DA model, as well as the variables with p|corr| value > 0.58 in S-plot, and the variables with confidence interval crossed zero in jack-knifed loading plot were considered as potential biomarkers. The potential biomarkers were identified by using Masslynx (Waters Technologies, United States) combined with the METLIN database^1^ and HMDB database^2^. Then, receiver operating characteristic (ROC) curves (SPSS 19.0) were applied to analyze data for evaluating the predictive power of the identified biomarkers. Pathway analysis was depended on KEGG database^3^, MetaboAnalyst 3.0^4^.

### Statistical Analysis

Individual data was expressed as Mean ±_standard deviation (SD). Statistical analyses were performed using one-way ANOVA (version 13.0, SPSS). Statistically significant changes were classified as significant (^∗^) where *p <* 0.05, more significant (^∗∗^) where *p <* 0.01 and highly significant (^∗∗∗^) where *p* < 0.001.

## Results

### ZJP Possesses Anti-diabetic Activity

Before drug treatment, ZJP was firstly chemically standardized. An HPLC-MS method was developed to reveal the profile of ZJP extract and quantify the main ingredients in the extract. By using the respective individual standard, ten chemical markers were identified from ZJP extract (Supplementary Figure 1) and the minimum amount in mg/g of dried extract was 0.0070 for β-ecdysone, 0.1163 for calycosin 7-O-glucoside, 0.0366 for acteoside, 0.1901 for naringin, 0.4649 for notoginsenoside R1, 0.1567 for ginsenoside Re, 0.7127 for ginsenoside Rg1, 0.4280 for icariin, 0.8445 for ginsenoside Rb1, 0.1868 for astragaloside IV and 0.0633 for schisandrin. The chemical analysis of ZJP extract here served as the quality control for the reproducibility of the below animal study.

**FIGURE 1:**
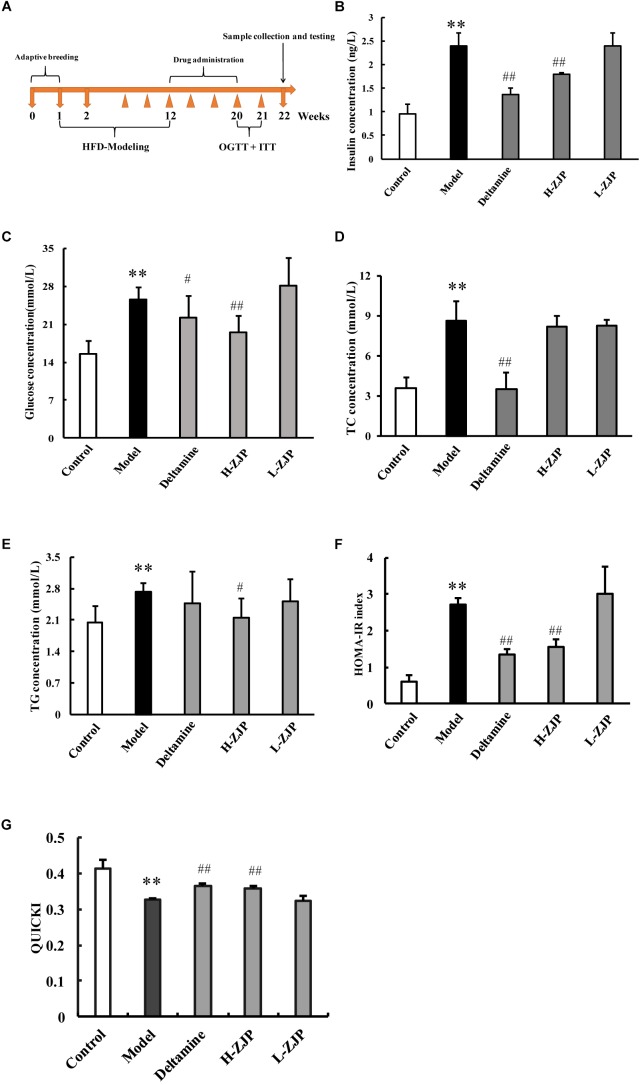
Effects of ZJP on biochemical indexes in diabetic mice. **(A)** Schedule of study process. **(B)** Fasting blood insulin test result. **(C)** Fasting blood glucose test result. **(D)** Blood total cholesterol concentration. **(E)** Blood triglycerides concentration. **(F)** Insulin resistance index (HOMA-IR). **(G)** Quantitative insulin sensitivity check index (QUICKI) test result. Data are presented as the means ± SEM, *n* = 6 mice per group. (^∗∗^*P* < 0.01 compared with the control group; ^#^*P* < 0.05, ^##^*P* < 0.01 compared with the model group).

To investigate the anti-diabetic efficacy of ZJP on animal model, the diabetic mice induced by high-fat diet were employed for this study. The timeline of current study is presented in Figure 1A. After drug administration for 8 weeks, OGTT and ITT were performed to confirm the efficacy of drug treatment (Supplementary Figure 2). Compared with normal group, mice from the high-fat diet group showed increased levels of serum insulin, glucose, and total cholesterol by ∼2.5-fold, 1.6-fold, and 2.4-fold, respectively (Figure 1B–D). The increased levels of insulin, glucose and total cholesterol could be significantly decreased by high dose of ZJP treatment compared with model group, and ZJP treatment at low dose showed some improvement but not in a significant level (Figure 1B–D). The triglyceride levels did not show obvious change in both model and drug treatment groups (Figure 1E). In addition, the insulin resistance index (HOMA-IR) and quantitative insulin sensitivity check index (QUICKI) were calculated. The HOMA-IR and QUICKI results further supported the improvement of high-fat diet group by drug treatment (Figure 1F,G). Deltamine treatment served as a positive control, which could improve insulin sensitivity as compared to the diabetic group.

**FIGURE 2:**
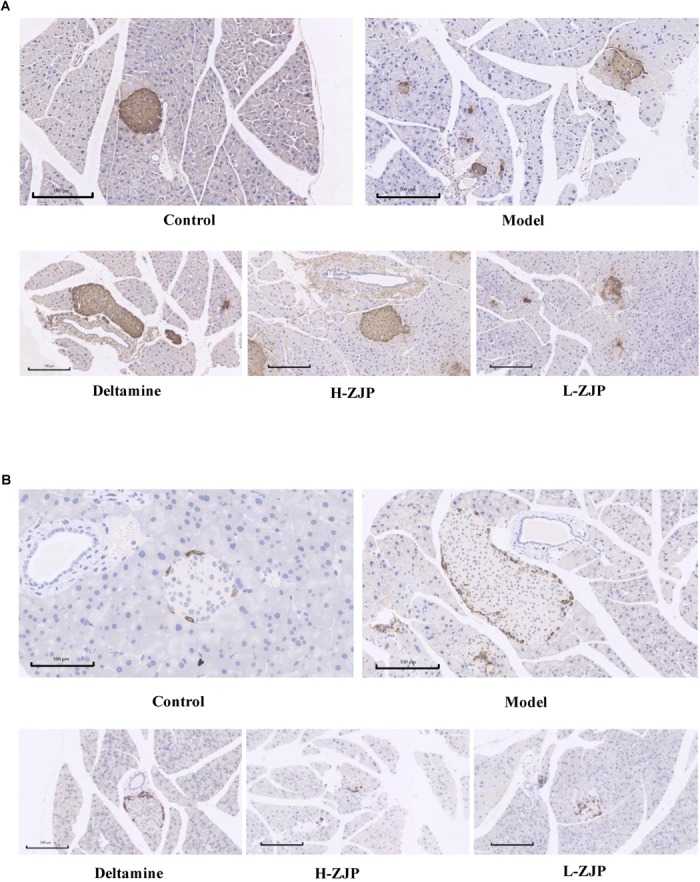
Immunohistochemistry analysis of pancreatic islets. Pancreatic islet tissues of mice were stained with insulin **(A)** and glucagon **(B)**. Representative images are shown at identical magnification, ×200, scale bar = 100 μm.

Immunohistochemical analysis demonstrated that pancreatic islets in model mice exhibited strikingly different appearance, with poor insulin staining and increased glucagon staining, and that of ZJP treatment mice were showed to be improved compared with model group (Figure 2A,B).

### The Repeatability and Stability for UHPLC-Orbitrap/MS Analysis

The overlapping total ion current (TIC) chromatograms (Supplementary Figure 3) of QC samples showed acceptable variations occurred during large-scale sample analysis. Meanwhile, six selected extracted ion chromatograms (EICs) in QC samples were employed to evaluate system repeatability and stability. Taking sample in positive mode as an example, retention times, peak areas and mass accuracies of these six selected peaks demonstrated acceptable RSDs. RSDs of these six peaks were 0.036–0.088% for retention times, 3.25–15.60% for peak areas, and 0.8 × 10^−4^%–1.0 × 10^−4^% for mass accuracies (Supplementary Tables S1, S2).

**FIGURE 3:**
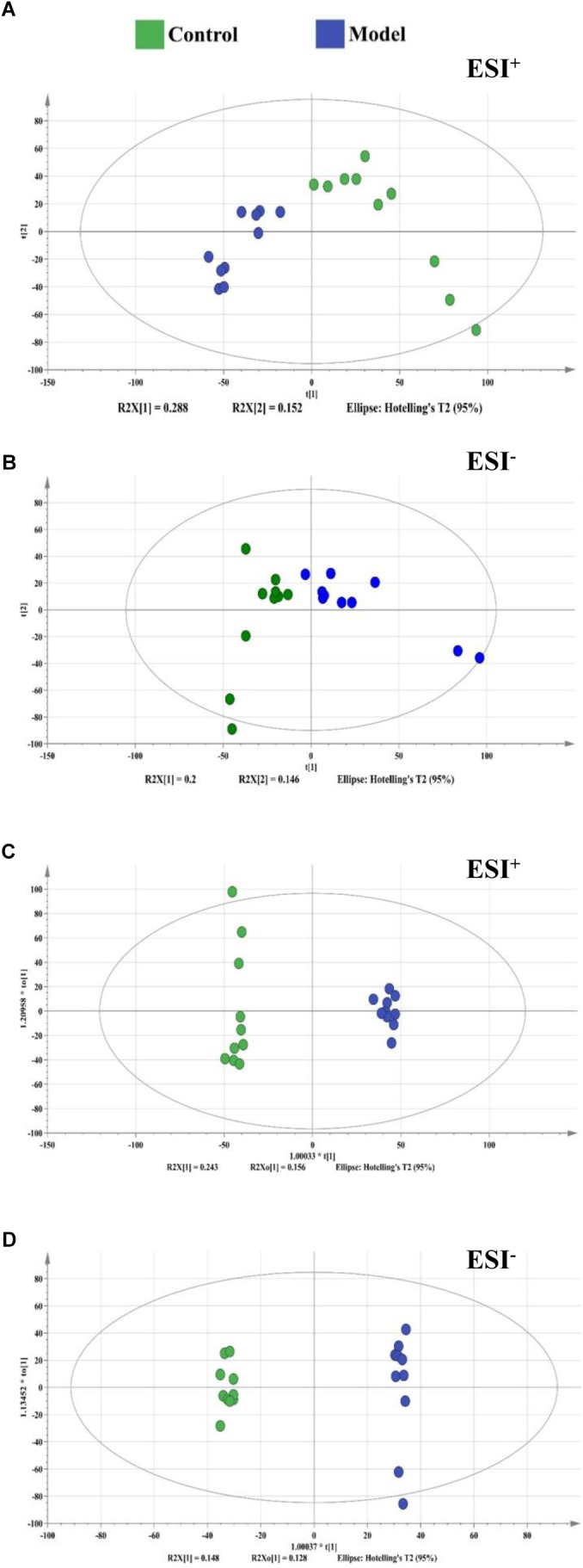
The score plots from PCA and OPLS-DA model based on metabolic profiling. **(A)** PCA-X score plot of positive ion mode. **(B)** PCA-X score plot of negative ion mode. **(C)** OPLS-DA score plot of positive ion mode. **(D)** OPLS-DA score plot of negative ion mode. ESI^+^ means the positive electrospray ionization. ESI^−^ means the negative electrospray ionization. Blue dots represent control group, green dots represent model group.

### UHPLC-Orbitrap/MS Metabolic Profiles of Normal and Diabetic Mice

By using the developed UHPLC-Orbitrap/MS method, a total of 8000/8000 peaks (ESI^+^/ESI^−^) were detected in serum samples. The vectors of data from control and model groups were collected as a matrix for PCA and OPLS-DA analysis to discover potential variations. The score plots of PCA demonstrated a clear separation between control and model groups in the metabolic profiles (Figure 3A,B). Similar findings were found in the score plots of OPLS-DA (Figure 3C,D). For OPLS-DA analysis, the model showed excellent classification and predictive ability between control and model groups with the intercept R^2^Y above 0.99 and *Q*^2^ above 0.85 (Figure 3).

### Potential Biomarkers for High-Fat Diet Induced Diabetes

**FIGURE 4:**
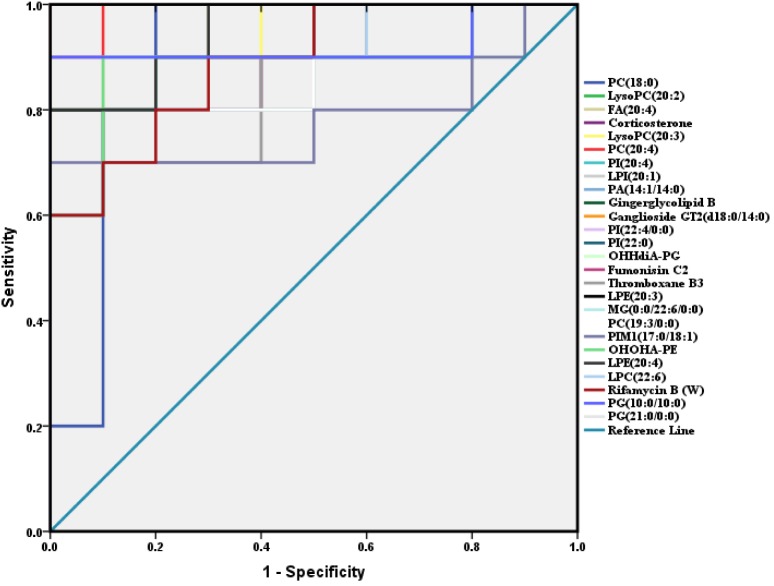
ROC analysis of 26 potential biomarkers from serum in diabetic mice. The detailed area under the curve (AUC), *p*-value, 95% confidence interval and the error value of the 26 identified potential biomarkers are indicated in the ROC curve. The 26 up regulated metabolites provided good diagnostic abilities with average area under the curve at 0.78–1.00 and *p*-value at 0–0.034.

A total of 26 potential biomarkers were found and identified in plasma samples (Table 2). The ROC curve analysis demonstrated the detailed area under the curve (AUC), *p*-value, 95% confidence interval and the error value of the 26 identified potential biomarkers for diabetes prediction (Figure 4). The 26 metabolites showed excellent diagnostic properties with average area under the curve at 0.78–1.00 and *p*-value at 0–0.034. Here, the data indicated that the identified biomarkers exerted a powerful diagnostic ability.

**Table 2:** Identities of differential metabolites between control and model.

NO.	Abbreviation	Molecular formula	m/z	RT (min)	Matched Mass	ppm	VIP	*P*-value	Fold
1	PIM1 (17:0/18:1)	C_27_H_50_NO_7_P	1051.5714	15.06	1051.573	−1.6	1.81	0.0063	2.75
2	LPC (22:6)	C_31_H_51_NO_9_P	612.3296	12.31	612.3307	−1.8	3.98	0.0000	1.86
3	PG (10:0/10:0)	C_26_H_51_O_10_P	553.3109	12.81	553.3147	−6.9	1.75	0.0001	1.47
4	PC (20:4)	C_28_H_50_NO_7_P	566.3199	14.44	566.3217	−3.2	2.62	0.0000	3.36
5	PI (20:4)	C_29_H_49_O_12_P	603.2886	15.6	603.2929	−7.1	1.76	0.0011	1.64
6	OHOHA-PE	C_30_H_54_NO_10_P	664.3003	12.84	664.3067	−9.6	3.73	0.0017	1.21
7	Fumonisin C2	C_33_H_57_NO_14_	692.3841	8.67	692.3852	−1.6	3.59	0.0000	5.62
8	LysoPC (20:2)	C_28_H_54_NO_7_P	570.3502	15.93	570.3530	−4.9	3.53	0.0004	1.90
9	LPE (20:4)	C_25_H_44_NO_7_P	500.2768	12.85	500.2783	−3.0	7.23	0.0001	1.39
10	PC (18:0)	C_26_H_55_NO_7_P	524.3682	18.42	524.3711	−5.5	1.20	0.0003	1.70
11	PG (21:0/0:0)	C_27_H_55_O_9_P	589.3329	13.52	589.3278	8.7	2.97	0.0000	4.39
12	OHHdiA-PG	C_31_H_58_NO_13_P	684.3685	8.99	684.3719	−5.0	3.56	0.0000	3.51
13	MG (0:0/22:6/0:0)	C_25_H_38_O_4_	385.2707	15.07	385.2737	−7.8	1.40	0.0034	1.93
14	LPI (20:1)	C_29_H_56_O_12_P	627.3471	8.64	627.3504	−5.3	1.31	0.0000	4.14
15	Thromboxane B3	C_20_H_32_O_6_	432.2360	13.29	432.2357	0.7	1.26	0.0068	1.14
16	PI (22:0)	C_31_H_60_O_13_P	671.3724	8.67	671.3766	−6.3	2.22	0.0000	6.04
17	Gingerglycolipid B	C_33_H_58_O_14_	679.3900	8.41	679.3899	0.1	1.39	0.0000	9.65
18	PC (19:3/0:0)	C_27_H_50_NO_7_P	549.3703	16.57	549.3663	7.3	1.06	0.0038	1.57
19	LPE (20:3)	C_25_H_46_NO_7_P	504.3040	15.7	504.3085	−8.9	1.41	0.0023	1.76
20	LysoPC (20:3)	C_26_H_54_NO_7_P	546.3508	17.92	546.3554	−8.4	1.22	0.0010	1.73
21	FA (20:4)	C_20_H_32_O_2_	305.2455	19.08	305.2475	−6.6	1.38	0.0002	1.61
22	LPI (20:1)	C_29_H_56_O_12_P	627.3471	8.64	627.3504	−5.3	1.31	0.0000	4.14
23	PI (22:4/0:0)	C_31_H_52_O_11_P	631.3311	8.64	631.3242	10.9	3.15	0.0000	5.57
24	Corticosterone	C_21_H_30_O_4_	347.2199	9.29	347.2217	−5.2	1.62	0.0009	1.55
25	Ganglioside GT2 (d18:0/14:0)	C_86_H_150_N_4_O_42_	645.3298	8.99	645.3254	6.8	2.16	0.0000	4.57
26	Rifamycin B (W)	C_39_H_49_NO_14_	740.2934	14.3	740.2924	1.4	1.90	0.0012	1.73

*VIP, variable importance in the projection. P-value was obtained from Wilcoxon–Mann–Whitney test.*

### ZJP Induces Metabolic Change of Experimental Mice

**FIGURE 5:**
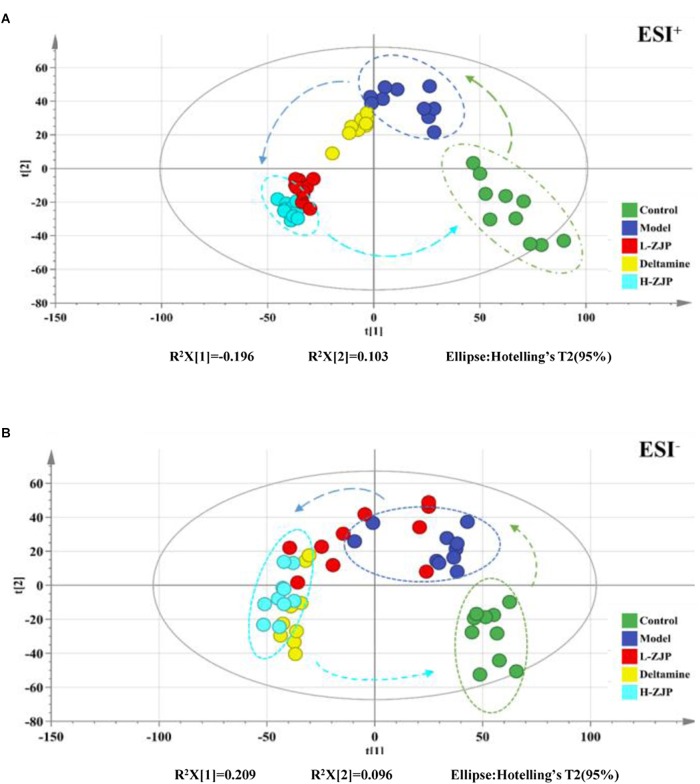
Effect of ZJP on the metabolic profiling by PLS-DA score plots. PLS-DA score plot of positive ion mode **(A)** and negative ion mode **(B)** for the control, model, and treated groups.

**FIGURE 6:**
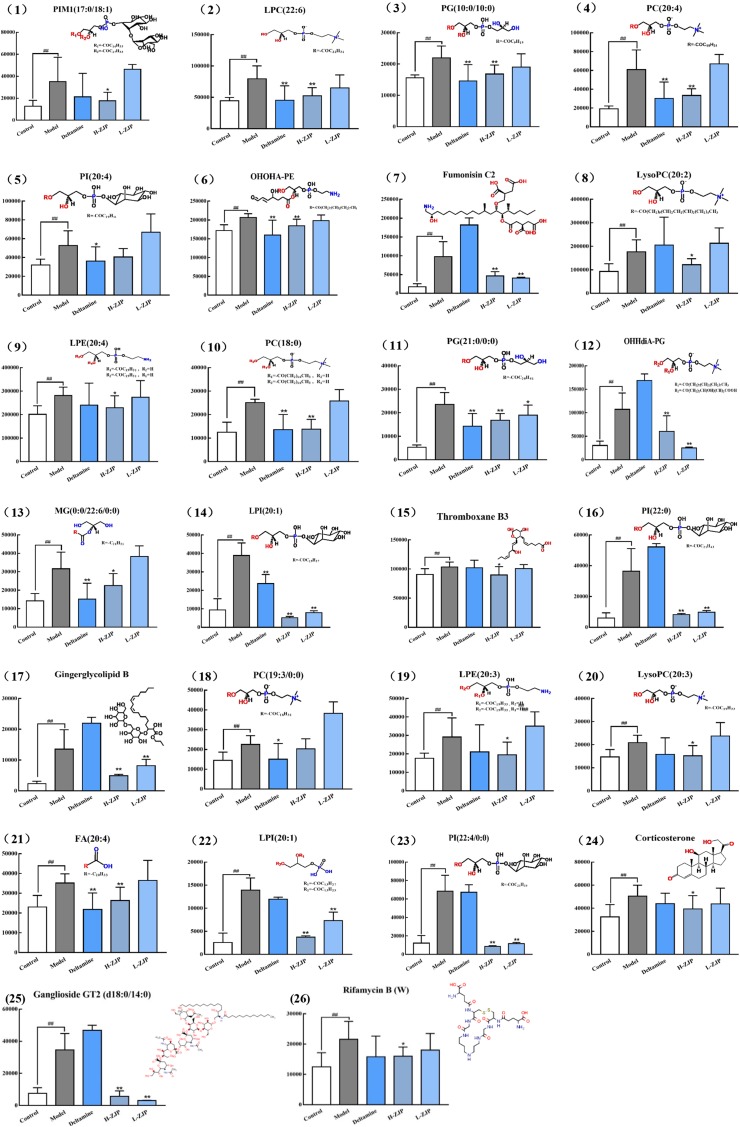
The relative content of 26 metabolites. The metabolites were identified in serum sample. Data are represented as means ± SD (*n* ≥ 8, ^∗^*P* < 0.05, ^∗∗^*P* < 0.01 vs. control; ^##^*P* < 0.01 vs. model).

In order to reveal the beneficial role of ZJP for treating diabetes, PLS-DA analysis was conducted to obtain the changes of metabolic trajectory after drug treatment. As indicated in Figure 5, metabolic trajectory of model group was gradually being moved toward to that of control group after administration of ZJP or deltamine. Moreover, the relative quantities of 26 potential metabolites were obtained using peak intensities from the resultant data matrices, amongst which 24 metabolites were robustly affected by ZJP compared with model group (Figure 6). Here, ZJP treated-mice restored to normal levels altered by the high-fat diet, which included down regulation of biomarkers.

### Metabolomics Pathway Analysis

The aforesaid databases in Method Section were used for analyzing the 26 metabolites related metabolic pathways. The topology map analysis described the impact of 26 metabolites between control and model groups on metabolic pathway. As indicated in Supplementary Figure 4, these metabolites were mainly involved in glutathione metabolism, steroid hormone biosynthesis and glycerophospholipid metabolism. These three principal pathways therefore were suggested to be closely involvement in the anti-diabetic action mechanism of ZJP.

## Discussion

Type 2 diabetes mellitus is well characterized by insulin resistance, often related to obesity, and impaired insulin secretion that eventually result in hyperglycemia and its associated complications. Finding out risk factors likely to progress to T2DM will help target these intervention approaches (Lowe and Bain, 2013). It is widely accepted that many diseases are relevant to alterations of metabolic profiling. Metabolomics has shown great promise to discover disease specific metabolic signatures and potential biomarkers. Studies utilizing metabolomics to seek for potential biomarkers have increased dramatically over the past years, which can provide insight into pathways important for T2DM pathogenesis. A group of biomarkers may be helpful to understand the key features of T2DM, and be useful for the prevention, diagnosis, and treatment of T2DM (Friedrich, 2012). Metabolomics nowadays is commonly employed for revealing the therapeutic effects and mechanisms of TCM (Wang et al., 2015, 2017). In this study, an UHPLC-Orbitrap/MS-based metabolomics approach was conducted to explore the potential mechanisms of ZJP in the treatment of T2DM. We found that ZJP treatment decreased the blood glucose level in high-fat diet diabetic mice. In support of this notion, previous findings have showed that ZJP improved the glucose tolerance in diabetic rats by using the OGTT (Li et al., 2018).

Increasing evidences support that obesity is an undisputed risk factor for T2DM, and nearly half of adult diabetics are considered obese (Nguyen et al., 2011). The high-fat fed mice is a commonly used model to study obesity-related T2DM (Eisinger et al., 2014; Saravanan and Pari, 2015; Yang et al., 2018). In this study, we observed that high-fat diet mice have increased body weight and glucose level. Moreover, the serum triglycerides were not altered significantly, while cholesterol showed to be notably increased. Our findings are consistent with the results from previous reports (Eisinger et al., 2014). Our metabolomic analysis revealed that model and control groups were obviously discriminated from each other on PLS-DA score plot. Major metabolites contributing to the discrimination were lipid metabolites including phospholipids, lysophospholipids and fatty acids, which were positively related to obesity-associated diabetes. In agree of this notion, Kim et al. (2011) found that a high-fat diet increased lipid metabolites. Furthermore, the levels of these metabolites were significantly down regulated by ZJP treatment.

Dietary phosphatidylcholine (PC) intake can raise the risk of T2DM (Li et al., 2015). In addition, studies suggest that those who consume more amount of PC have higher rates of all-cause and cardiovascular-specific mortality in the United States population, especially in patients with diabetes (Zheng et al., 2016). In this study, we noted that high-fat diet induced the increase of PC (22:6), PC (20:4), PC (18:0) and PC (19:3/0:0) levels in mice serum, and ZJP treatment reduced the above PCs levels except for PC (19:3/0:0).

Lysophosphatidylcholine (LysoPC), the absorbed form of PC, has been considered to be an important lipid intermediate that links saturated fatty acids, such as palmitic acid (PA), to insulin resistance. Han et al. found that LysoPC content was increased in the liver and muscle of db/db mice, which was suppressed by bromoenol lactone, one of inhibitors of calcium-independent phospholipase A_2_ that blocks the insertion of PA to LysoPC (Han et al., 2011). Moreover, LysoPC was thought to be a PKCζ activator (Siddiqi and Mansbach, 2015). Chen et al. reported that inhibition of PKCζ prevented glucocorticoid-induced glucose intolerance in mice (Chen et al., 2017). Taken together, we may assume that the increased LysoPC levels could activate PKCζ which leads to inhibitory serine phosphorylation of insulin receptor substrate 1 (IRS1) and ultimately glucose uptake. In support of this, in this study, ZJP treatment markedly reversed high-fat diet induced increases in LysoPC levels, i.e., LysoPC (20:2) and LysoPC (20:3) in mice. However, Barber et al. (2012) revealed there was a reduction in LysoPC concentrations in obesity and T2DM. Besides, LysoPC (18:2) was reported to be associated with decrease risk of T2DM (Floegel et al., 2013), and lower level of LysoPC was found to be predictors for T2DM (Wang-Sattler et al., 2012). Therefore, further study is needed to investigate the functions of lysoPC species in states of diabetes.

In our findings, we also observed that a high-fat diet exhibited high ganglioside GT2 concentration and a marked reduction by ZJP treatment. In agreement with this, a report showed that inhibition of ganglioside synthesis resulted in improved insulin sensitivity and enhanced activatory tyrosine phosphorylation of IRS1 in the muscle (Ritter et al., 2015). Zayed et al. (2018) found that the levels of phosphatidylinositol (PI) in diabetic patients were higher than that of non-diabetic patients. Our metabolomics profile revealed that the obese diabetic mice significantly increased the levels of PI (20:4), PI (20:0), PI (22:4/0:0). Furthermore, ZJP restored the increased PI (20:0) and PI (22:4/0:0) levels, and showed a tendency to down regulate PI (20:4).

Glutathione metabolism pathway has been suggested to be closely related to high-fat diet induced diabetic mice in present study. Glutathione deficiency contributes to oxidative stress, which plays a key role in the pathogenesis of diabetes (Wu et al., 2004). A high-fat diet lowered the NAD/NADH ratio, suggesting the irregular lipid and energy metabolism (Kim et al., 2011; Yamato et al., 2016). The level of glutathione is reduced and the proper antioxidant may be often insufficient in diabetic conditions (Agarkov et al., 2014). Thus, normalization of the glutathione antioxidant system and NAD/NADH ratio could be useful approaches for treating obesity or diabetes.

## Conclusion

In conclusion, we demonstrated herein that ZJP treatment exhibited anti-diabetic effect on high-fat diet-induced obese diabetic mice, the effect of which might be due to the regulation of phospholipid metabolism. Although some metabolites have been dug out in this study, it is still needed to be further confirmed whether these metabolites are equivalent in the ZJP pharmaceutical function by the MS data. A major challenge for the future is to characterize the individual functions of the altered metabolites circulating in blood to elucidate the deep mechanism of ZJP on diabetes disease. Besides, numerous metabolite changes found in mouse model also needs to be validated by using patient samples. A prospective follow-up study may provide additional insight into these limitations.

## Author Contributions

HL and JC contributed to concept, design, literature search, and manuscript review. JC, ZH, and LZ contributed to lab work, analysis, and interpretation of data. PZ, SZ, and TY contributed to definition of intellectual content and manuscript review. FW, LZ, and SH contributed to lab work, acquisition of data, and drafting the manuscript. All authors have read and approved the manuscript.

## Conflict of Interest Statement

The authors declare that the research was conducted in the absence of any commercial or financial relationships that could be construed as a potential conflict of interest.

## Footnotes

1 https://metlin.scripps.edu

2 http://www.hmdb.ca

3 http://www.genome.jp/kegg

4 http://www.MetaboAnalyst.ca/
