# Supplementary material for: Metabolomics Reveals Effect of Zishen Jiangtang Pill, a Chinese Herbal Product on High-Fat Diet-Induced Type 2 Diabetes Mellitus in Mice
Source: Front Pharmacol. 2019 Mar 19;10:256. doi: 10.3389/fphar.2019.00256 (PMC6434817; doi:10.3389/fphar.2019.00256)
Supplement: Supplementary file 1 [file Data_Sheet_1.pdf]

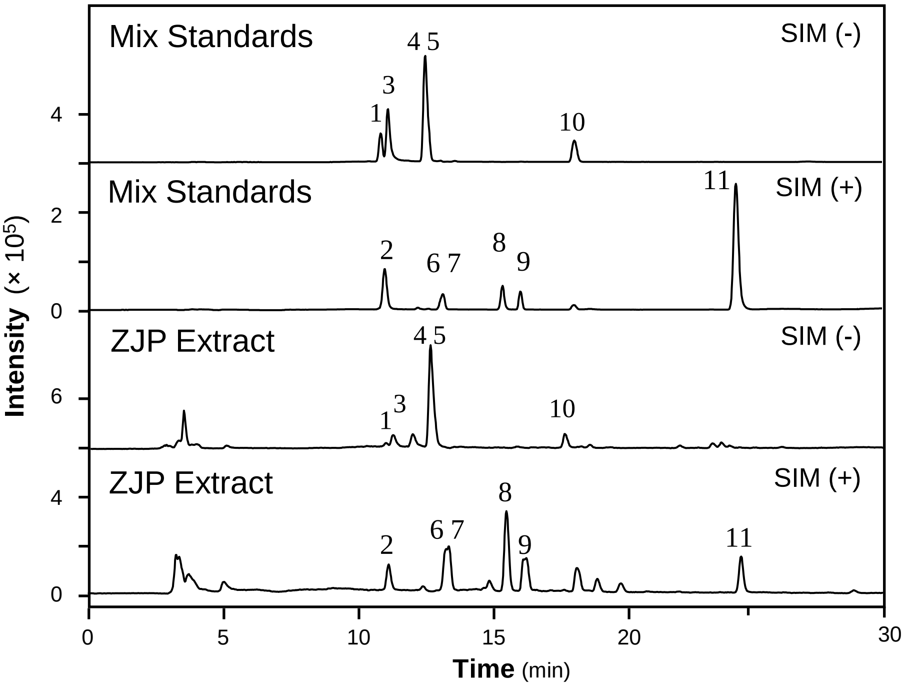

Supplementary Figure 1. HPLC-MS chromatogram of ZJP extract.

Equipment type: Shimadzu LC-20A; chromatographic column: Agilent 5TC-C18 (250 mm×4.6 mm, 5 μm); flow rate: 0.8 ml/min; mobile phase condition: the proportion of water (A) and acetonitrile (B) was as follows: 0-25 min ,19%-85%B；25-30 min, 85%-19%B.. A Shimadzu mass spectrum (LC-2020) equipped with an electrospray ionisation (ESI) ion source was operated in positive and negative modes, and the selected ion monitoring (SIM) was used. Optimized mass spectra were acquired with an interface voltage of 4.5 kV. Nitrogen was used as nebulizer gas at a flow rate of 1.5 L/min and dry gas flow of 15 L/min. Shimadzu Mass workstation software was used for data acquisition and processing. The denotation peaks 1-11 were *β*-ecdysone (1), calycosin 7-O-glucoside (2), acteoside (3), naringin (4), notoginsenoside R1 (5), ginsenoside Re (6), ginsenoside Rg1 (7), icariin (8), ginsenoside Rb1 (9), astragaloside IV (10) and schisandrin (11). Representative chromatograms are shown, n = 3.

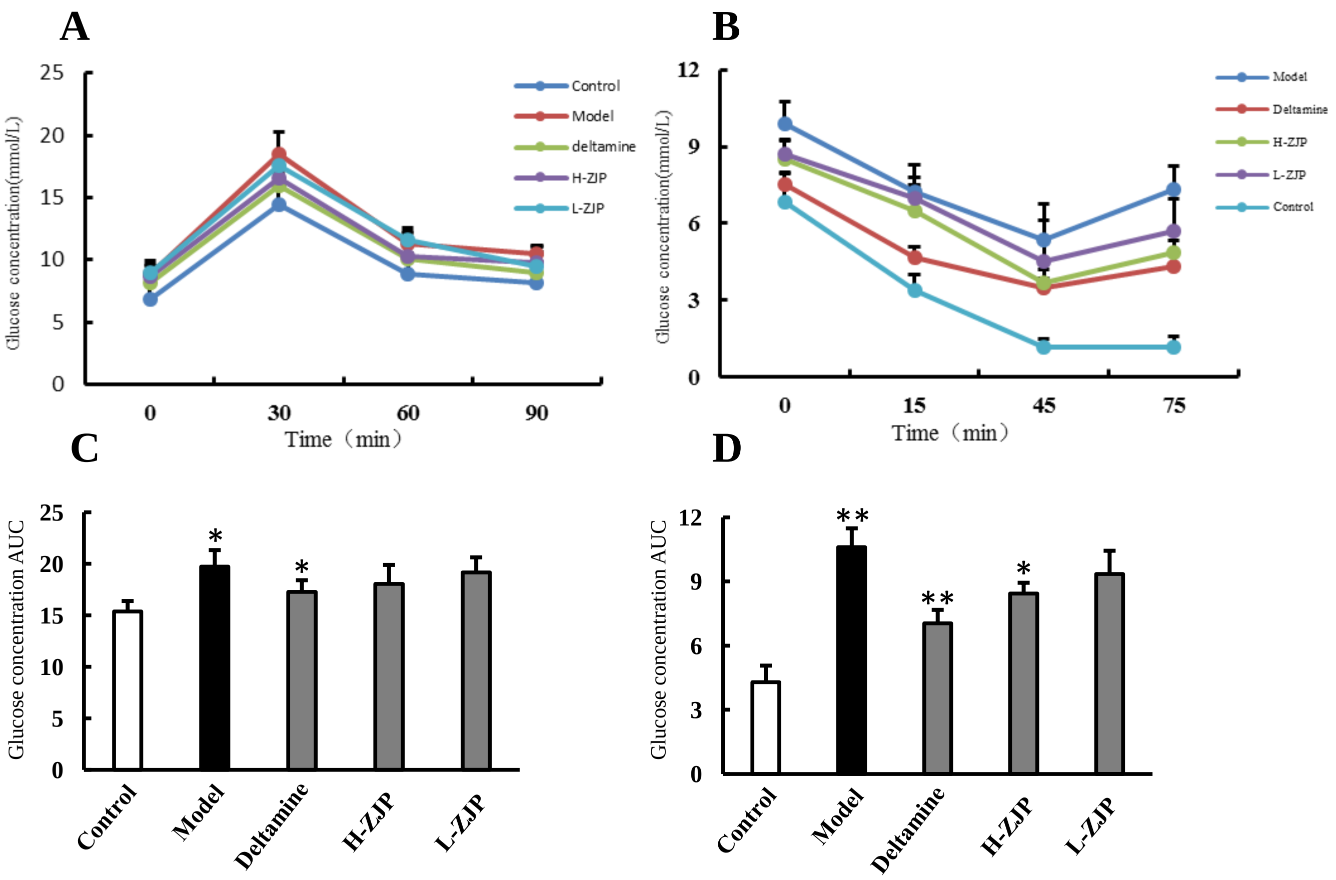

Supplementary Figure 2. OGTT and ITT results after 8 weeks drug administration.

(A) oral glucose tolerance test result; **(B)** insulin tolerance test result. **(C)** AUC of oral glucose tolerance test; **(D)** AUC of insulin tolerance test. The area under the blood glucose curve (AUC) is calculated by the following formula:

$AUC = 0.5 \times [BgV1 + BgV2] / 2 + 0.5 \times [BgV2 + BgV3] / 2 + 1 \times [BgV3 + BgV4] / 2$, where V1, V2, V3, V4, and V5 were blood glucose values at different time points.

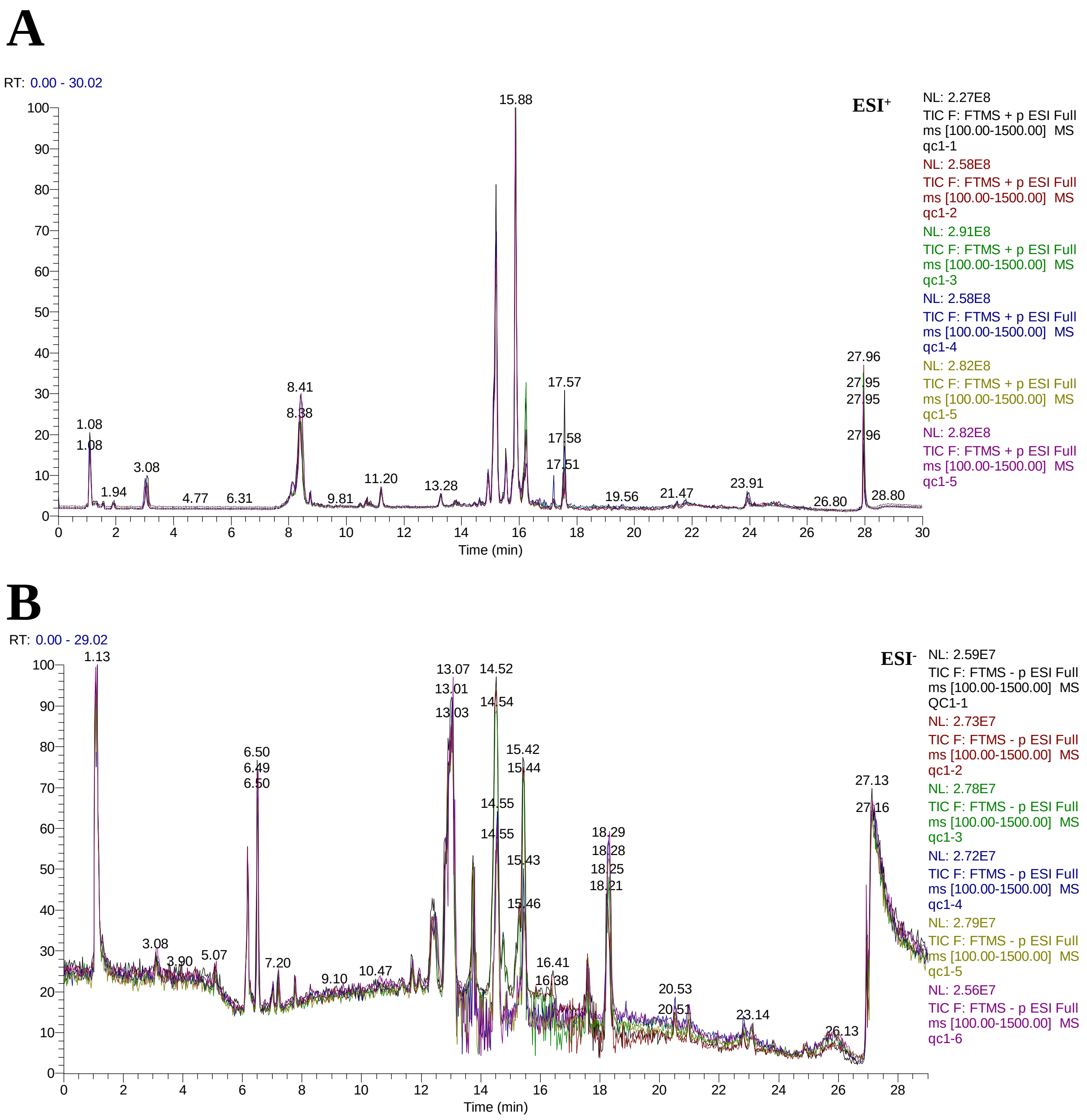

Supplementary Figure 3. The overlapping typical total ion chromatograms (TICs) of QC samples obtained from LC-MS.

(A) Six QC samples from serum in positive mode. **(B)** Six QC samples from serum in negative mode.

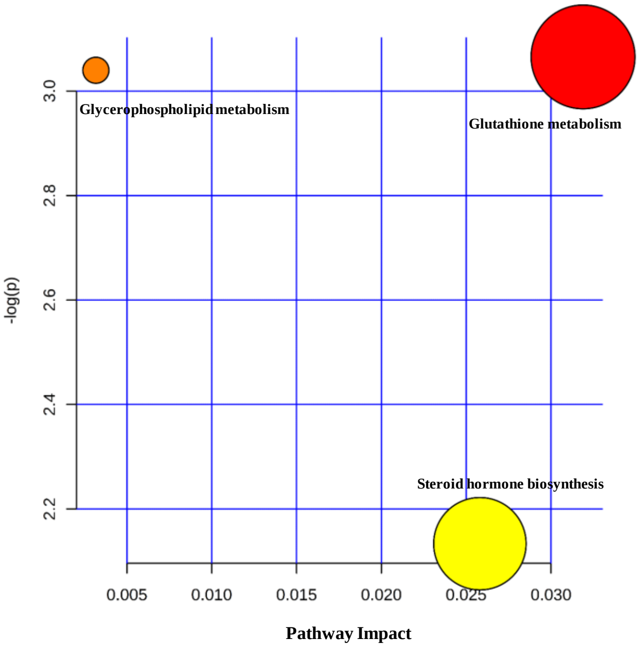

Supplementary Figure 4. Disordered pathways in model group.

The topology map generated from Metabo Analyst 3.0 (http://www.MetaboAnalyst.ca/) indicated the impact of 26 metabolites between control and model groups on metabolic pathway. Small p-value and big pathway impact factor revealed the pathway is significantly affected.
