# Supplementary material for: Metabolomics Reveals Effect of Zishen Jiangtang Pill, a Chinese Herbal Product on High-Fat Diet-Induced Type 2 Diabetes Mellitus in Mice
Source: Front Pharmacol. 2019 Mar 19;10:256. doi: 10.3389/fphar.2019.00256 (PMC6434817; doi:10.3389/fphar.2019.00256)
Supplement: Supplementary file 2 [file Data_Sheet_2.pdf]

TABLE S1. Six extracted ions in ten QC samples from serum in positive mode.

Extracted Ions	No.	Retention time	Peak area	Mass accuracies
524.3682	1	17.54802	374345	524.36737
	2	17.52866	348961	524.36774
	3	17.54261	481511	524.36700
	4	17.56247	398180	524.36676
	5	17.5456	313976	524.36658
	6	17.54665	328328	524.36975
	7	17.54638	296649	524.36700
	8	17.56587	322327	524.36615
	9	17.5545	403297	524.36639
	10	17.58532	324047	524.36707
	RSD	0.08836	15.60301	1.9×10^{-4}
546.3508	1	15.75870	733397	546.35205
	2	15.75916	722333	546.35260
	3	15.74710	744170	546.35144
	4	15.74313	696637	546.35144
	5	15.74594	747450	546.35138
	6	15.74398	715262	546.35211
	7	15.76272	730296	546.35162
	8	15.75604	711530	546.35107
	9	15.75133	668538	546.35120
	10	15.74847	716509	546.35168
	RSD	0.04453	3.25028	0.9×10^{-4}
566.3199	1	15.20587	42593	566.31726
	2	15.20766	40637	566.31738
	3	15.19577	42638	566.31628
	4	15.19333	42346	566.31647
	5	15.19644	43013	566.31641
	6	15.19181	41663	566.31708
	7	15.19356	34330	566.31641
	8	15.20370	45304	566.31604
	9	15.19999	44394	566.31622
	10	15.19581	42790	566.31677
	RSD	0.03684	7.10215	0.8×10^{-4}
504.304	1	15.69795	31500	504.30566

	2	15.69866	28993	504.30606
	3	15.71761	26874	504.30505
	4	15.7158	27730	504.30518
	5	15.71744	27465	504.30475
	6	15.71447	26728	504.30566
	7	15.70638	27749	504.30533
	8	15.72637	27885	504.30463
	9	15.7230	27431	504.30447
	10	15.71811	27522	504.30521
	RSD	0.06106	4.92619	1.0×10^{-4}
385.2707	1	14.85185	29005	385.27292
	2	14.85416	28501	385.27322
	3	14.84176	28872	385.27243
	4	14.83997	29532	385.27249
	5	14.84210	31842	385.27255
	6	14.83863	23516	385.27310
	7	14.84731	28588	385.27264
	8	14.85036	28916	385.27222
	9	14.84632	30213	385.27237
	10	14.84180	29013	385.27271
	RSD	0.03619	7.31530	0.8×10^{-4}
1051.5714	1	15.06453	26344	1051.57202
	2	15.06649	28299	1051.57361
	3	15.0546	28316	1051.57092
	4	15.05213	27056	1051.57092
	5	15.05510	25985	1051.57141
	6	15.0508	26485	1051.57251
	7	15.05271	23849	1051.57190
	8	15.06254	27165	1051.57019
	9	15.05866	29141	1051.57080
	10	15.05481	28169	1051.57082
	RSD	0.03667	5.63915	1.0×10^{-4}

TABLE S2. Six extracted ions in ten QC samples from serum in negative mode

Extracted	No.	Retention time	Peak	Mass accuracies
664.3002	1	12.79821	198356	664.30096
	2	12.79695	195622	664.30066
	3	12.79954	199293	664.30176
	4	12.79891	196705	664.30029
	5	12.79878	197616	664.30133
	6	12.77010	225530	664.30109
	7	12.82923	225350	664.30090
	8	12.80378	228426	664.30019
	9	12.79594	203162	664.30054
	10	12.81934	197724	664.30028
	RSD	0.12094	6.64083	0.8×10^{-4}
500.2773	1	12.74202	259884	500.27792
	2	12.76729	255349	500.27689
	3	12.77136	262471	500.27765
	4	12.76924	254651	500.27798
	5	12.76928	250829	500.27777
	6	12.74059	266546	500.27789
	7	12.82923	266637	500.27811
	8	12.79828	277407	500.27656
	9	12.77039	261994	500.27788
	10	12.80713	255063	500.27810
	RSD	0.21711	2.98601	1.0×10^{-4}
612.33	1	12.27436	60076	612.33002
	2	12.27362	59634	612.32971
	3	12.27587	60796	612.33044
	4	12.27507	58035	612.33026
	5	12.27512	57779	612.33014
	6	12.24593	53905	612.32971
	7	12.33439	54772	612.33008
	8	12.31534	56041	612.33343
	9	12.27890	60728	612.32989
	10	12.30378	56009	612.33010
	RSD	0.20660	4.34748	1.8×10^{-4}
740.2931	1	14.27003	22715	740.29388

	2	14.27995	21980	740.29407
	3	14.28303	21904	740.29382
	4	14.28324	21815	740.29321
	5	14.28195	22762	740.29340
	6	14.25460	27429	740.29358
	7	14.31406	24759	740.29279
	8	14.30156	27276	740.29378
	9	14.27560	20734	740.29402
	10	14.30904	20927	740.29267
	RSD	0.12726	10.51324	0.7×10^{-4}
553.3121	1	12.79821	18472	553.31250
	2	12.76950	17983	553.31238
	3	12.77136	18727	553.31238
	4	12.74074	19144	553.31262
	5	12.74095	18028	553.31201
	6	12.71193	17657	553.31226
	7	12.80056	18045	553.31256
	8	12.79467	18757	553.31234
	9	12.75329	17763	553.31247
	10	12.79245	18212	553.31215
	RSD	0.23536	2.62856	0.3×10^{-4}
589.3327	1	13.49253	12236	589.33319
	2	13.49145	12352	589.33368
	3	13.49537	11853	589.33398
	4	13.49374	11920	589.33313
	5	13.49378	11512	589.33295
	6	13.46476	13930	589.33356
	7	13.43672	14365	589.33301
	8	13.49358	14754	589.33324
	9	13.49475	11442	589.33374
	10	13.48356	10537	589.33321
	RSD	0.14117	11.13177	0.6×10^{-4}
